# Climate change-informed dietary modeling in Pacific cod: Experimentally-derived effects of temperature and dietary quality on carbon and nitrogen stable isotope trophic discrimination factors

**DOI:** 10.1371/journal.pone.0295564

**Published:** 2023-12-07

**Authors:** Matthew C. Rogers, Ron A. Heintz, Johanna J. Vollenweider, Ashwin Sreenivasan, Katharine B. Miller

**Affiliations:** 1 NOAA, National Marine Fisheries Service, Alaska Fisheries Science Center, Auke Bay Laboratories, Juneau, Alaska, United States of America; 2 Sitka Sound Science Center, Sitka, Alaska, United States of America; 3 University of Alaska Southeast, Juneau, Alaska, United States of America; Universidad de Cadiz Facultad de Ciencias del Mar y Ambientales, SPAIN

## Abstract

Stable isotope analysis is a powerful tool for dietary modeling and trophic ecology research. A crucial piece of information for isotopic dietary modeling is the accurate estimation of trophic discrimination factors (TDFs), or the isotopic offset between a consumer’s tissue and its diet. In order to parameterize stable isotope dietary models for future climate scenarios, we investigated the effect of water temperature and dietary protein and lipid content on TDFs in juvenile Pacific cod (*Gadus macrocephalus*). Pacific cod are a commercially and ecologically important species, with stock numbers in the northeast Pacific recently having dropped by more than 70%. We tested four water temperatures (6, 8, 10, and 12°C) and two dietary regimens (low and high lipid content), representing a range of potential ocean temperature and prey quality scenarios, in order to determine carbon and nitrogen TDFs in juvenile Pacific cod. Additionally, we assessed dietary intake and proximate composition of the experimental fish in order to estimate consumption, assimilation, and retention of dietary nutrients. The results of this study suggest that dietary protein catabolism is a primary driver of nitrogen TDF variability in juvenile Pacific cod. Across all temperature treatments from 6 to 12°C, fish reared on the lower quality, lower lipid content diet had higher nitrogen TDFs. The mean TDFs for fish raised on the higher lipid, lower protein diet were +3.40 ‰ for nitrogen (Δ^15^N) and +0.36 ‰ for lipid-corrected carbon (Δ LC ^13^C). The mean TDFs for fish raised on the lower lipid, higher protein diet were +4.09 ‰ for nitrogen (Δ^15^N) and 0.00 ‰ for lipid-corrected carbon (Δ LC ^13^C). Lipid-corrected carbon isotope data showed that, regardless of temperature, fish consuming the lower lipid diet had essentially no trophic discrimination between diet and bulk tissues. We found no ecologically meaningful differences in TDFs due to water temperature across the 6°experimental range. The results of this experiment demonstrate that dietary quality, and more specifically the use of dietary protein for energetic needs, is a primary driver of trophic discrimination factors. The TDFs determined in this study can be applied to understanding trophic ecology in Pacific cod and closely related species under rapidly changing prey availability and ocean temperature conditions.

## Introduction

The past five decades have seen steady growth in the use of stable isotope data for understanding linkages in food webs, consumers’ trophic position, and seasonal variation of consumer diets [1–6]. As stable isotope data have become more accessible and common, so have stable isotope mixing models for estimating proportions of different prey items in consumers’ diets. The newest generation of Bayesian stable isotope mixing models incorporate multiple sources of uncertainty and are more robust in estimating diet proportions from numerous isotopically distinct sources than previous generations of models [7]. A crucial piece of the stable isotopic dietary modeling puzzle is the accurate estimation of trophic discrimination factors (TDFs), or the isotopic offset between a consumer’s tissue and its diet.

Trophic discrimination factors in dietary modeling studies have often been estimated based on averages from many different taxa, often under a broad umbrella of similarity (e.g. ‘carnivores’, ‘aquatic species’) [8]. Studies focusing on TDF variability and its consequences, however, have shown that TDFs vary by species, tissue type, diet quality, and other factors [9–11]. Species-specific TDFs are preferable when possible, although performing controlled feeding trials for a full range of taxa is difficult, and sometimes it is impossible for species that cannot be raised in captivity. Many studies still utilize TDFs from closely related taxa or generic TDFs from metaanalyses, and when using non-species specific TDFs in an isotopic diet modelling study, the need for additional information such as tissue type, trophic position, and diet source becomes crucially important [11]. Controlled experiments to determine isotopic TDFs are therefore extremely valuable when possible for testing a range of variables that may affect TDFs both within and across species [12].

Trophic discrimination factors have been experimentally determined for many species over the course of the past few decades, and these studies have been compiled in two large-scale meta-analyses investigating the drivers of TDFs across taxa, within species, and between tissues [8, 11]. Fish species are well represented in these studies. Stephens et al. [11] found, however, that most fish studies investigating diet by using stable isotope mixing models use broadly generic TDF values from literature that are not species or even taxa specific. For marine species, a minority of TDF studies (approximately one in five) evaluate the effect of environmental temperature on trophic discrimination [10, 11] and even fewer incorporate both temperature and dietary quality [11, 13, 14]. Water temperature has been shown in some cases to effect nitrogen TDFs; in one study Δ^15^N values decreased by 1.6% as temperature increased from 4 to 27°C, while water temperature effects on carbon isotope trophic discrimination are unclear [10]. Higher diet quality has generally been shown to negatively affect TDFs, with a higher quality diet consisting of enough energy density to satisfy maximal growth and maintenance demands, as well as high quality protein content that matches or exceeds the specific amino acid profile required by a given species and tissue [11, 15]. Despite the work that has been done to date studying the drivers of TDF variability, we still lack a lack an understanding of how these factors mechanistically influence TDF values along with a comprehensive framework to account for them, particularly for diet composition [11]. Both of these factors, temperature and diet quality, are likely to be affected in the long term as mean ocean temperatures steadily rise, and more acutely during periods of prolonged extreme warming in ocean waters, also known as marine heatwaves.

In late 2013, a marine heatwave developed in the northeastern Pacific Ocean that increased sea surface temperatures by more than four standard deviations at Ocean Station Papa in the Gulf of Alaska [16], and the mass of warm water persisted into 2016 [17]. During this marine heatwave, he abundance of low lipid zooplankton increased [18, 19] while the abundance of small, lipid-rich copepods (e.g. *Pseudocalanus* sp.) became more variable. The result was an overall reduction in the abundance of lipid-rich prey [20].This reduction in food quality in concert with elevated temperatures led to poor survival among larval gadids [21, 22], decreases in forage fish abundance and their lipid content [23, 24] and collapse of some commercial fisheries [25]. Pacific cod were particularly affected, with a 71% decrease in adult biomass between 2015 and 2017 [26], resulting in an emergency closure of the Gulf of Alaska cod fishery in 2018.

Marine heatwaves are expected to become longer and more frequent in the coming decades [27] with concomitant biotic effects such as decreases in biomass, shifts in biogeography, and changes in the quality of prey for commercially important fish species [28]. Understanding how TDFs in marine fish respond to both ocean water temperature and dietary quality will be crucial to performing accurate dietary modelling exercises in changing climate scenarios, particularly for marine heatwaves.

In order to parameterize stable isotope dietary models for future climate scenarios and better understand the mechanisms driving TDF variability, we investigated the effect of temperature and dietary quality on TDFs in Pacific cod. We performed a fully factorial feeding trial in Pacific cod with four temperature treatments in the likely range of future ocean temperatures in the Gulf of Alaska in the coming decades [29], and with two experimental diets–lower and higher lipid content–that simulated the range of dietary lipids in potential Pacific cod prey. We also measured food intake, assimilation, and growth in order to determine if dietary protein or dietary lipid content affected TDFs. We calculated TDFs (Δ^15^N, Δ^13^C, and lipid corrected Δ^13^C) for all treatments and determined drivers of TDF variability in Pacific cod.

## Methods

### Collection and culture

Newly settled age-0 Pacific cod were collected in beach seines on Brothers Island in southeastern Alaska (57.2985 N -133.8254 W) on June 9, 2019. Captured fish were transported to the Auke Bay Laboratories facility in Juneau, Alaska, transferred to a 1500 L tank, and quarantined for 30 days at approximately 8°C. On July 9, 2019, all captured fish were divided among 16 tanks; approximately 25 fish were randomly chosen for each 400 L tank. Tanks were then divided into four temperature treatments. Four tanks were assigned to each temperature treatment of 6, 8, 10, or 12 ⁰C. The water supplying each group of four tanks was adjusted to the target temperature over the next 15 days. During the quarantine and temperature adjustment periods, fish were fed a pelleted commercial diet (BioVita, BioOregon, Washington USA).

Beginning July 26, hereafter referred to as day 0, fish in two of the tanks within each temperature treatment were offered a high lipid diet and the remaining two tanks from the same temperature treatment were offered a low lipid diet. Each treatment level (Temperature × Diet) had two replicate tanks in order to account for potential tank effects. Food was offered *ad libitum* once each day. The food for each tank was weighed on a wet mass basis before feeding, and after half an hour the uneaten food was siphoned out and weighed to determine consumption rates. Fish were fed daily until day 56 (September 20, 2019) at which point the fish had reached approximately three times their initial mass.

#### Diets

Diets consisted of homogenized mixtures of water, Pacific herring (*Clupea pallasii*), squid, euphausiids, supplemental vitamins, and gelatin. The homogenized mixture was supplemented with corn oil to create the high lipid diet or dry fish protein to create the low lipid diet. Each ingredient was procured in sufficient quantities at the outset of the experiment to last through the duration of the experiment in order to maintain isotopic consistency. Batches of each diet were prepared throughout the experiment as needed and were tested for isotopic consistency. The target lipid levels in the two diets were based on reported differences in the lipid content of high latitude and middle latitude zooplankton and the range of lipid content of typical diet items of Pacific cod during the recent past in the Gulf of Alaska [30–32]. Samples of the diets were retained at three dates (Day 0, Day 27, Day 50) during the experiment to determine the proximate composition, energy density, and isotopic composition and to ensure energetic and isotopic consistency. Analysis of proximate composition and isotopic composition followed procedures outlined below. The high lipid diet, on a wet mass basis, was 4.8 kJ/g, 13.2% protein and 6.0% lipid, while the low lipid diet was 5.6 kJ, 20.5% protein and 1.5% lipid. The isotopic composition of the high lipid diet was δ^15^N = 8.16 ± 0.15 ‰, δ^13^C = -20.48 ± 0.28 ‰, and lipid corrected δ^13^C = -17.76 ± 0.15 ‰. For the low lipid diet, the isotopic composition was δ^15^N = 8.19 ± 0.11 ‰, δ^13^C = -17.75 ± 0.08 ‰, and lipid corrected δ^13^C = -16.27 ± 0.05 ‰. Dietary lipid correction was performed on the final composed dietary samples for each diet treatment using the marine fish-specific formula outlined in Kiljunen et al. [33], as the final composition of the diet on a dry mass basis was more than 50% fishmeal in both treatments.

### Sampling & proximate composition

On day 15 when fish were moved to experimental tanks, and experimental days 0, 19 (August 14), 31 (August 26), and 56 (September 20), the fish were inventoried, anesthetized using tricaine methanesulfonate (MS-222), measured for total length (± 1 mm) and weight (± 0.01 g), and returned to their tanks. Food was withheld for one day prior to each sampling day to minimize stress during handling. At the end of the experiment, all of the surviving fish were sacrificed for proximate composition and bulk isotopic analysis. Whole individual fish were homogenized and aliquots were taken to determine their moisture, protein, lipid and isotopic content. The goal of the proximate analysis was to determine how ingested energy was allocated while the goal of isotopic analysis was to determine trophic discrimination factors (TDFs) for fish fed each of the different diets. Proximate composition (moisture, ash, lipid, and protein content) was determined following the methods outlined fully in Rogers et al. [34].

### Food intake and assimilation

The goals of measuring temperature and food quality effects on assimilation, metabolism, and growth required estimation of the daily consumption, daily assimilation, and daily changes in fish size. Assimilated material is the consumed material that remains after losses due to excretion and fecal production. Estimates of consumption and assimilation were estimated for each day fish were fed the experimental diets. Estimates of growth relied on observed changes in the average size on the days in which tanks were censused. The bioenergetics model is described in detail by Deslauriers et al. [35].

Daily consumption rates were estimated for each tank on each day as grams of wet food consumed per gram of fish. Daily consumption rates were adjusted to account for fecal production and urinary excretion [36] and the dry mass of the food to determine the dry mass assimilated as g of dry food assimilated per dry g of fish. Multiplying the total dry mass assimilated in the tank by the energy density of the food and dividing by the tank’s dry biomass provided daily estimates of the energy assimilated (kJ per gram dry mass per day).

Estimates of consumption and assimilation rates required estimation of the wet weight and dry weight of the fish in each tank on each day. Observations of fish wet weights on days 0, 19, 31, and 56 were used to provide estimates for the intervening days by fitting each day to a regression relating the mean wet mass observed in a tank on each of the 4 sampling dates. The average percent moisture, percent lipid, and percent protein were estimated for each tank on days 15, 24, and 56. The percent moisture for a fish for each day of the study was estimated by fitting a linear regression model for each tank. The model provided an estimated percent moisture for each fish in each tank. The product of the average wet mass and one minus the proportion that was moisture provided daily estimates of the average fish dry mass. Similarly, it was necessary to estimate the lipid and protein content of the fish on day 0 of the experiment. Observations of the average percent lipid in each tank were linearly regressed on sampling dates (days 15, 24, and 56). Day 0 was fit to the resulting model. A similar procedure was employed to estimate the average protein content of fish on day 0. The average dry mass and lipid content of the fish on days 0 and 56 were estimated from their proximate compositions.

### Isotopic analysis & trophic discrimination factors

Dried samples of homogenized whole fish tissue and each of the diets were examined to identify their isotopic compositions. Individual whole fish homogenate was utilized for this experiment as many studies employ whole fish homogenates when analyzing small or juvenile fish due to the ease of homogenization [37–39]. Additionally, Schielke and Post [40] advocated for the use of whole-body isotopic composition in smaller individuals since muscle plug samples do not accurately represent the whole-body isotopic composition of smaller juvenile individuals. Approximately 1.0 mg of ground whole fish tissue was weighed into tin capsules for analysis. Bulk stable isotopic analysis was performed using a FlashSmart elemental analyzer in line with a Delta V continuous-flow isotope ratio mass spectrometer (Thermo Scientific, Waltham, Massachusetts, USA) at the Auke Bay Laboratories in Juneau, Alaska, USA. Stable isotope values are reported in delta (δ) notation relative to international standards (vs. Air for δ^15^N and vs. VPDB for δ^13^C). The instrument was calibrated using certified reference materials from the International Atomic Energy Agency and the US Geological Survey. Internal laboratory standards (purified methionine and homogenized Chinook salmon [*Oncorhynchus tshawytscha*] muscle) were used as quality controls and yielded long-term precision estimates of ± 0.13 ‰ for nitrogen and ± 0.12 ‰ for carbon.

We calculated trophic discrimination factors (TDF) for nitrogen (Δ^15^N), carbon (Δ^13^C), and lipid-corrected carbon isotope data (LC Δ^13^C) using the difference between mean isotopic values from homogenized whole fish tissue and the mean isotopic values of food. TDF values were calculated according to the equation: Δ_TISSUE_ = mean(δ_TISSUE_−δ_FOOD_) where Δ_TISSUE_ represents the tissue- and isotope-specific TDF, *δ*_TISSUE_ is the isotope value of for the whole body of each animal, and *δ*_FOOD_ is an average isotope value of the food. Carbon isotope data are presented for both non-lipid corrected and lipid-corrected fish tissue values in order to facilitate all modeling efforts commonly performed by the stable isotopic mixing model community. Lipid corrections made to carbon isotope data were performed using the marine organism-specific formula outlined in Kiljunen et al. [33]. We used the ‘lmer’ function in the ‘lme4’ package (Version 1.1–34) to perform a linear mixed-effects model in R (Version 4.1.1, R Core Team) to determine if there were significant differences in TDFs between treatments due to temperature, diet, or the interaction of the two. All data were tested for assumptions of the linear mixed effects model, and Diet and Temperature were set as fixed effects and Tank was set as a random effect. Model comparisons were made using AIC and the model with the lowest AIC score was selected. We then used a Tukey’s HSD post-hoc test in the ‘lsmeans’ R package (Version 1.3–5) to determine which treatment means were significantly different by pairwise comparisons (S1 File).

### Ethics statement

The National Marine Fisheries Service’s Animal Care and Use Policy (04–112) does not include any requirements for research on captive or wild fish. Fish used in this study were collected and handled in accordance within the guidelines of the U.S. Government Principles for the Utilization and Care of Vertebrate Animals Used in Testing, Research, and Training (https://olaw.nih.gov/policies-laws/phs-policy.htm, accessed on 5/2/2023) and the American Fisheries Society Guidelines for the Use of Fishes in Research (https://fisheries.org/docs/policy_useoffishes.pdf; Chapter V, accessed on 5/2/2023). This work was completed under The Alaska Department of Fish and Game, United States permit CF-21-034. This permit allows the capture and transport of Pacific cod to the National Marine Fisheries Service laboratory in Juneau, Alaska, United States, and for subjects to be held on site. No species listed as threatened were captured. Reports on catch and disposition were delivered and reviewed for permit compliance by the Alaska Department of Fish and Game.

## Results

### Fish culture, proximate composition & trophic discrimination factors

Fish increased in length by 39% and increased in weight by 192% over the course of the experiment (Table 1). On day 0, fish averaged 2.6 g ± 0.1 g and 67.3 mm ± 0.5 mm in total length (All error is reported as S.D.). After 56 days, all individuals averaged 7.6 g ± 0.2 g and 93.8 mm ± 1.0 mm in length. Temperature was the only factor to influence this growth and its effect was marginal (P = 0.065). There was no evidence of an effect of diet on length or weight (P = 0.214) or the interaction between diet and temperature (P = 0.441). Fish fed either diet assimilated similar dry masses of diet, roughly ~2.5 g per fish over 56 days. However, the dry mass composition of the diets differed. Fish consuming the low lipid diet assimilated 2.47 g ± 0.18 g of protein compared with 1.97 g ± 0.17 g for those on the high lipid diet (Table 1). The low lipid fish used much of this protein for energy, retaining about 25% or 0.61 g ± 0.06 g for tissue growth. In contrast, the high lipid diet group used lipid for energy allowing them to retain a greater proportion of the protein they consumed (36% or 0.70 g ± 0.10 g) for tissue growth.

**Table 1:** The average (± 1 s.e.) initial weight of fish held at different temperatures and fed different diets, the estimated average amount of dry mass they consumed over 56 days, and the mass retained as wet weight, dry material or lipid. Averages for each treatment were calculated from the mean value in each of two replicate tanks (i.e. n = 2).

Diet	Temperature	Initial weight (g)	Dry mass assimilated (g)	Final weight (g)	Change in dry mass (g)	Change in lipid mass (g)
**High Lipid**	6	2.2 ± 0.1	1.4 ± 0.08	5.4 ± 0.1	0.59 ± 0.05	0.24 ± 0.01
	8	2.4 ± 0.1	2.1 ± 0.10	8.2 ± 0.3	0.89 ± 0.37	0.42 ± 0.01
	10	2.9 ± 0.1	2.4 ± 0.41	8.6 ± 1.5	1.14 ± 0.01	0.40 ± 0.17
	12	2.8 ± 0.3	2.5 ± 0.44	10.0 ± 3.4	1.24 ± 0.16	0.49 ± 0.25
**Low lipid**	6	2.6 ± 0.1	1.5 ± 0.03	6.2 ± 0.1	0.60 ± 0.02	0.13 ± 0.01
	8	2.8 ± 0.1	1.9 ± 0.12	6.7 ± 0.6	0.62 ± 0.00	0.13 ± 0.04
	10	2.9 ± 0.1	2.2 ± 0.18	7.9 ± 0.4	0.71 ± 0.13	0.13 ± 0.01
	12	2.6 ± 0.0	2.4 ± 0.51	8.2 ± 1.6	0.79 ± 0.01	0.18 ± 0.05

**Fig 1:**
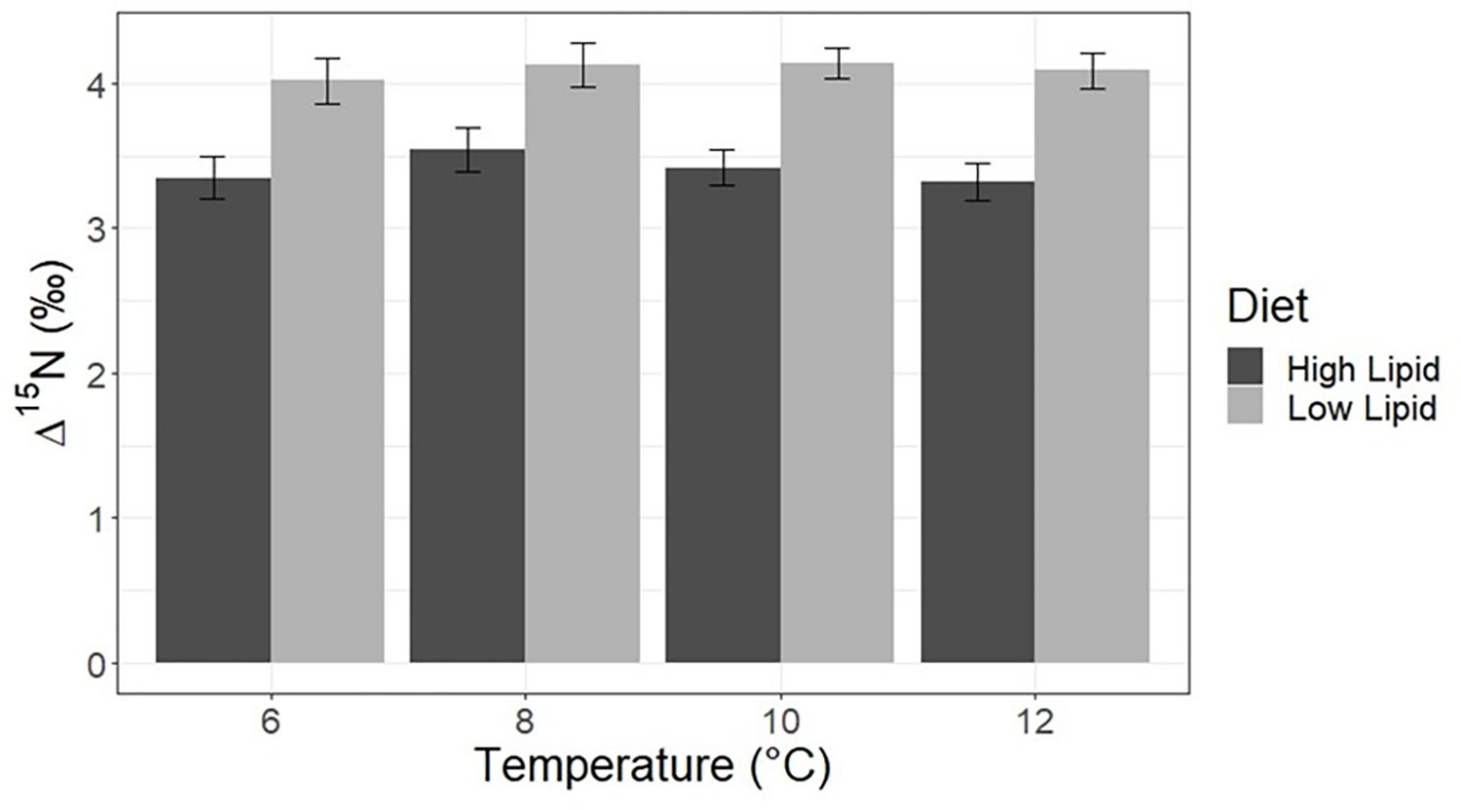
Nitrogen TDFs. Nitrogen stable isotope trophic discrimination factors for Pacific cod raised at four temperatures and on two diets of differing lipid contents. Error bars represent S.D.

**Fig 2:**
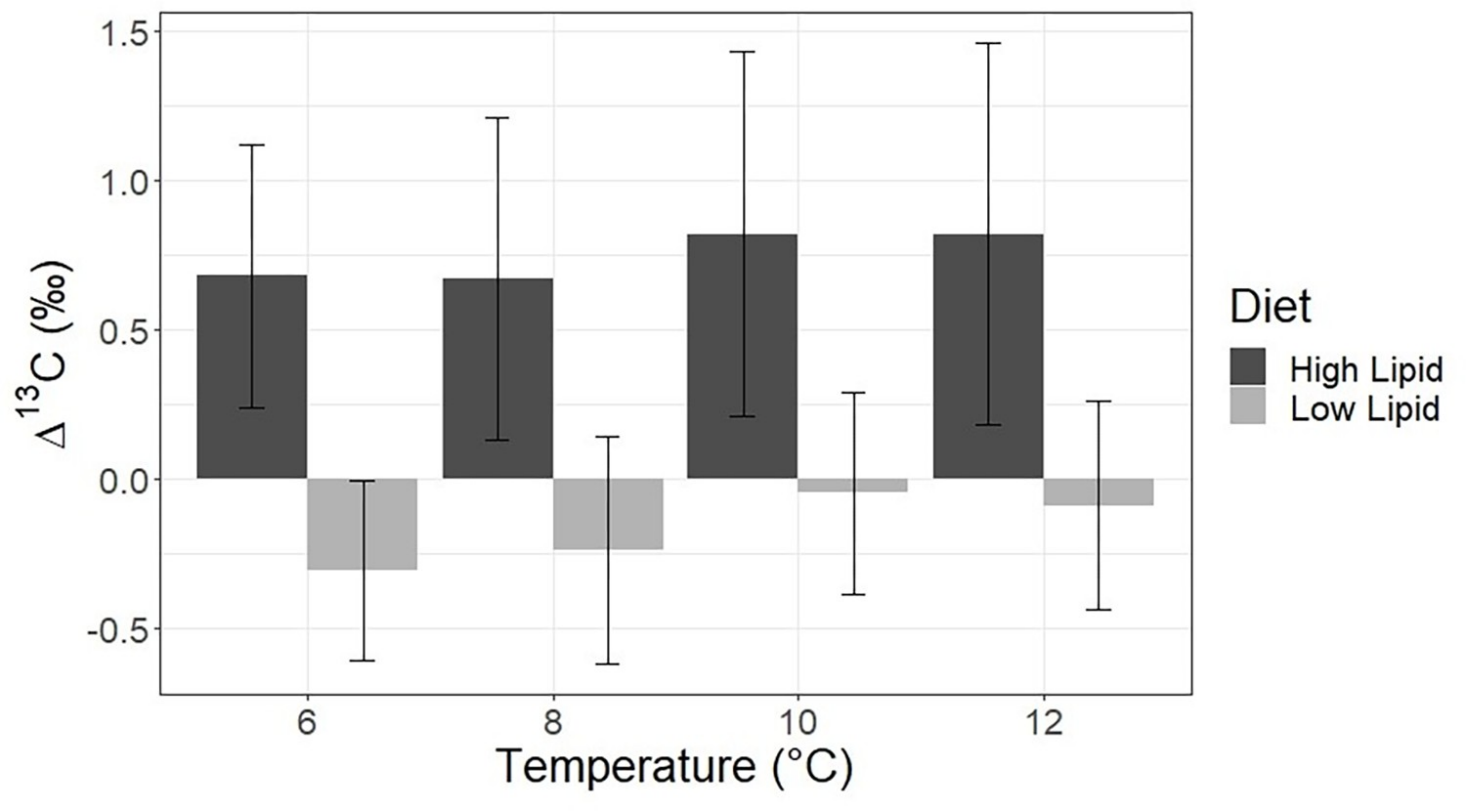
Carbon TDFs. Carbon stable isotope discrimination factors for Pacific cod raised at four temperatures and on two diets of differing lipid contents. Error bars represent S.D.

Diet quality, in terms of protein and lipid content, significantly and consistently affected ^15^N and ^13^C discrimination across all temperatures (Table 2). The mean ^15^N TDF (Δ^15^N) for fish on the low lipid diet was 4.09 ‰ +/- 0.14 ‰, while the mean ^15^N TDF for fish on the high lipid diet was 3.40 ‰ +/- 0.15 ‰. (Fig 1) There were very slight but statistically significant differences in Δ^15^N across temperatures within a dietary treatment (Table 1). The mean ^13^C TDF (Δ^13^C) for fish on the low lipid diet was -0.18 ‰ +/- 0.35 ‰, and the mean ^13^C TDF for fish on the high lipid diet was 0.75 ‰ +/- 0.57 ‰ (Fig 2). There were no statistically significant differences in Δ^13^C across temperatures within each dietary treatment.

**Table 2:** Stable isotopic trophic discrimination factors in juvenile Pacific cod for nitrogen, carbon, and lipid-corrected carbon isotopes. Pacific cod were fed either a higher lipid or lower lipid content diet and were raised at one of four temperatures (6,8,10, or 12°C) in a fully factorial experimental design. Superscripts denote statistical differences within an isotopic category (Δ^15^N, Δ^13^C, or Δ LC ^13^C) but not across categories.

	Temperature (°C)	N	Δ^15^N (‰) Mean	Δ^15^N (‰) S.D.	Δ^13^C (‰) Mean	Δ^13^C (‰) S.D.	Δ LC ^13^C (‰) Mean	Δ LC ^13^C (‰) S.D.
**High lipid**	All	92	3.32	0.20	0.93	0.65	0.43	0.23
	6	23	3.35^a^	0.15	0.68^a^	0.44	0.32^a^	0.15
	8	18	3.54^b^	0.15	0.67^a^	0.54	0.32^a^	0.19
	10	25	3.42^ab^	0.12	0.82^a^	0.61	0.38^a^	0.19
	12	26	3.32^a^	0.13	0.82^a^	0.64	0.41^a^	0.21
**Low lipid**	All	94	4.05	0.21	-0.18	0.33	-0.05	0.18
	6	27	4.02^c^	0.16	-0.31^b^	0.30	-0.03^bc^	0.12
	8	21	4.13^c^	0.15	-0.24^b^	0.38	-0.08^b^	0.15
	10	23	4.14^c^	0.11	-0.05^b^	0.34	0.03^bc^	0.11
	12	23	4.09^c^	0.12	-0.09^b^	0.35	0.07^c^	0.09

**Fig 3:**
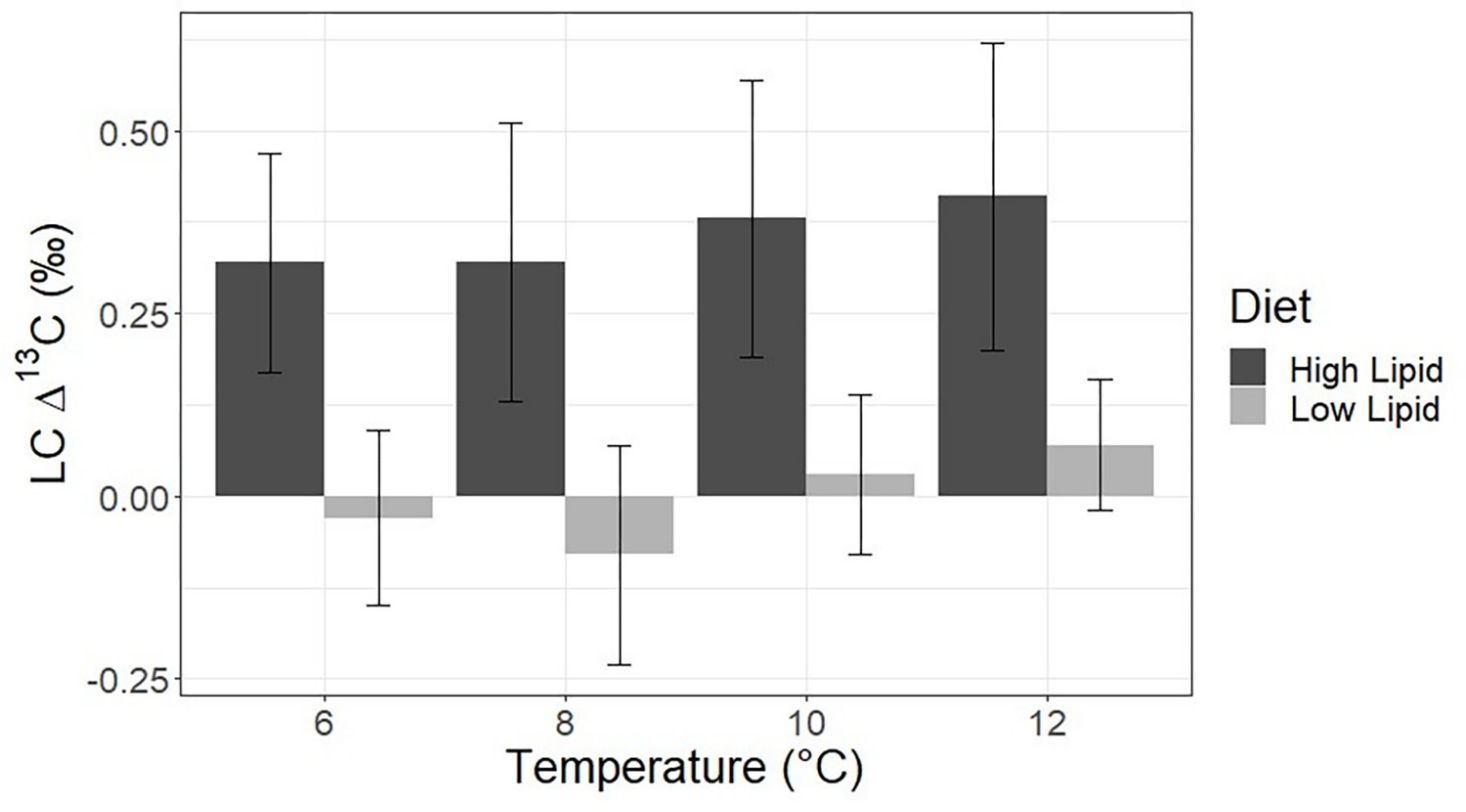
Lipid-corrected carbon TDFs. Lipid-corrected carbon stable isotope discrimination factors for Pacific cod raised at four temperatures and on two diets of differing lipid contents. Error bars represent S.D.

The mean lipid corrected ^13^C TDF (LC Δ^13^C) for fish on the low lipid diet was -0.00 ‰ +/- 0.13 ‰, and the mean lipid corrected ^13^C TDF for fish on the high lipid diet was 0.36 ‰ +/- 0.19 ‰ (Fig 3). There were very slight but statistically significant differences in LC Δ^13^C across temperatures within each dietary treatment.

## Discussion

### Nitrogen trophic discrimination factors

The results of this study show that dietary quality, as it influences lipid and protein catabolism, is a primary driver of TDF variability in juvenile Pacific cod. Nitrogen and carbon isotope TDFs were within the typical range of discrimination factors found in marine fish species, as summarized in a recent metaanalysis of TDFs across taxa [11]. Across all temperature treatments, fish raised on the lower lipid content diet had higher nitrogen TDFs (Δ^15^N –Fig 1,) which was likely driven by higher dietary protein catabolism to fuel energetic needs. This result is consistent with other studies of the relationship between dietary lipid and protein content and trophic discrimination factors in teleosts. Bloomfield et al. [13] determined that higher nitrogen trophic discrimination resulted from juvenile black bream (*Acanthopagrus butcheri*) eating a lower lipid, vegetable protein-based diet versus a higher lipid, fishmeal-based diet. Mohan et al. [41] and Britton and Busst [14] found higher nitrogen trophic discrimination due to lower dietary protein contents in controlled feeding studies of Atlantic croaker (*Micropogonias undulates*) and goldfish (*Carassius auratus*), respectively. Lastly, in a meta-analysis of dietary discrimination studies in teleosts, Canseco et al. [10] found higher Δ^15^N when fish were fed a lower quality pelleted algae/plant diet versus a raw fish or pelleted fishmeal diet.

The consistent differences in nitrogen TDFs due to dietary quality can most likely be attributed to the lower lipid diet not providing enough lipid to meet the fishes’ energetic requirements, and therefore requiring catabolism of dietary protein to meet the energetic needs of growth. McMahon et al. [42] demonstrated that two measures of dietary quality, protein content and amino acid imbalance, had significant negative relationships with nitrogen TDFs. By using compound-specific nitrogen isotopic analysis of amino acids, they determined that fish consuming a lower protein content diet with amino acid compositions that did not match their requirements had higher trophic discrimination factors. The likely primary mechanism for this relationship is increased deamination and transamination (‘protein scavenging’) during protein catabolism and synthesis in individuals that cannot meet metabolic requirements for particular amino acids, and the concurrent use of dietary protein to meet energetic needs [15, 42–44].

Previous studies have hypothesized that an increasing quantity of protein alone in a consumer’s diet leads to higher nitrogen TDFs [15, 45]. In many studies investigating the effect of dietary protein quantity on TDFs, however, the animals were fed *ad libitum* or at rations exceeding the maximal intake, and few of these studies have explicitly quantified the true protein consumption, assimilation, and catabolism of the individuals. Fish in this study assimilated the same amount of food on a dry mass basis. Fish on the higher lipid diet converted food into tissue more efficiently, and ended the experiment with a higher body lipid content than their initial state, while fish on the lower lipid diet had unchanged bodily lipid content from beginning to the end of the experiment. Fish from both treatments retained approximately the same mass of protein for tissue construction. Our results agree with the findings of Chikaraishi et al. [46], wherein tadpoles fed a high protein, low starch diet had large trophic amino acid nitrogen TDFs, which the authors hypothesized was due to the utilization of the bulk of dietary protein for energetic needs. Conversely, tadpoles fed a low protein, high starch diet had much lower nitrogen TDFs due to most of the animal’s energetic needs being met by starches, and the small amount of protein in the diet being routed directly to biomass production. Ultimately, our data suggest that fish on the lower lipid diet used dietary protein to a greater degree as energy to fuel growth, which resulted in greater dietary protein catabolism, with a concurrent increase in trophic discrimination for protein used in tissue growth.

In a broader, cross-species study of TDFs and dietary quality, Robbins et al. [15] found that protein quality (i.e., amino acid composition relative to requirements) was the primary driver across taxonomic classes in determining TDF magnitudes, while total protein intake relative to animal requirements accounted for variation within some subgroups. Nuche-Pascual et al. [47] conducted a meta-analysis over a broad range of protein levels (8–71%) in diets and found no relationship between amino acid TDFs and percent protein relative to nutritional requirements. Thus, the literature remains somewhat equivocal regarding the effects of protein quality and quantity on TDFs. Further detailed experimental work across a range of species, including Pacific cod, explicitly quantifying protein intake, species-specific amino acid requirements versus dietary amino acid composition, dietary lipid and energetic content, assimilated protein and energy, and macromolecule isotopic routing will help to refine knowledge of the precise mechanisms driving the relationships between dietary quality and TDFs. The results of this and other studies, however, indicate that one of the strongest links between diet and increased nitrogen TDFs is an increased level of amino acid catabolism from either diet or endogenous tissue and the resultant nitrogen isotope fractionations associated with deamination and transamination [47–50]. One potential line of research that has not been pursued to explore the drivers of TDFs across dietary quality regimens would be to measure aminotransferase enzymatic or transcription activity in fish fed diets with various levels of amino acid imbalances and various levels of dietary lipid.

The mean change in individual mass from the beginning to the end of the experiment (+ 192%) indicates that the vast majority of tissue built was composed of nutrients from the experimental diet and that the TDF estimates derived from the data are accurate. Additionally, tissue turnover rates in juvenile teleosts are variable but tend to be rapid, indicating that an additional proportion of tissue beyond newly added mass was constructed from the experimental diet. Martinez del Rio et al. [12] present a thorough summary of the effects of growth rate on isotopic incorporation. In young, fast-growing ectothermic animals, new tissue construction far outweighs tissue catabolism and thus drives isotopic incorporation [51, 52]. Scharnweber et al. [53] found strong relationships between metabolic rate and stable isotope TDFs (negative correlation for Δ^13^C and positive correlation for Δ^15^N) in Eurasian perch, with the smallest size classes (< 20 cm) having the highest metabolic rates. Precise tissue isotopic incorporation rates have not previously been determined for juvenile Pacific cod. Hesslein et al. [54] determined that the isotopic half-life of ^15^N in juvenile broad whitefish (*Coregonus nasus)* muscle was 9–22 days. Similar estimates have been found in other juvenile fish species [55]. Based on these estimates, the fish in this study had isotopic turnover of the majority of their active tissues during the study period in addition to the nearly 200% added mass from growth. Additionally, the condition of isotopic equilibrium is confounded in many situations in the wild where consumers such as Pacific cod may switch prey frequently. As such, these data represent TDF values for Pacific cod which are useful for real-world dietary scenarios and the TDFs are representative of individuals at or near isotopic steady state with their diet. In future studies, measuring stable isotope deposition in eye lenses [56] of sacrificed fish may help to determine if individuals have reached isotopic steady state with their diet, and potentially allow for fewer animals being sacrificed at intervals through a feeding trial.

A recent meta-analysis of TDFs across taxa has shown that dietary quality, trophic level and tissue type are significant drivers of TDF variability [11]. When these factors are accounted for, the possibility of using TDFs from one species for dietary stable isotope mixing models in another related species become more defensible. Many studies in fish species use broadly generic TDFs that do not account for dietary quality or prey type in their mixing model parameters [11]. The results of this work add to the body of knowledge about the relationship of diet to TDFs in Pacific cod specifically and marine teleosts generally, and allow for the TDFs derived herein to potentially be applied to other closely related species or taxa, with similar metabolic physiology and growth rates, as long as diet quality is taken into consideration [11].

### Carbon trophic discrimination factors

The lipid-corrected carbon isotope data showed that, regardless of temperature, fish consuming the lower lipid diet had essentially no trophic discrimination between diet and bulk tissues (Figs 2 and 3). Carbon isotope trophic discrimination factors determined in this study (approximately 0–1 ‰) are within the range of values observed in many other experiments, yet substantial variation can be found both across and within species due to factors such as dietary quality and tissue type [8, 11]. It is probable that fish consuming the lower lipid food utilized all of the lipid in their diet to fuel metabolic demands, essential tissue building, and growth [57], and did not have excess lipid to route to amino acid carbon side-chain construction for protein anabolism through secondary metabolism [58]. Fish on the high lipid diet, by contrast, had higher carbon trophic discrimination factors even after the data were corrected for lipid content (Fig 2). One potential explanation for this effect is that fish on the higher fat content diet had an excess of lipid in their diet beyond that needed for growth and metabolism and utilized lipids to a greater degree to help build muscle tissue, which has been well documented across species [59–62] through the processes of beta-oxidation and gluconeogenesis [63]. The increased level of breakdown of the carbon skeleton in lipids to refashion as the carbon constituents in protein, either in fish secondary metabolism or by gut microbiota [45, 64] could lead to greater carbon isotope fractionation compared to fish on the lower lipid diet and consequently a higher TDF for fish on the high lipid diet relative to the lower lipid diet. Because the supplemental lipid component of our experimental diet was vegetable oil from C_4_ plants, the δ^13^C value of the lipid fraction of the diet was enriched relative to typical marine fish lipid fractions [65, 66]. Future experiments including compound-specific isotopic analysis of tissue amino acids will help further elucidate the mechanisms of dietary lipid isotopic routing to proteinaceous tissues [61].

### Temperature effects on TDFs

We found very little difference in trophic discrimination factors across the experimental range of temperatures. Although there were statistically significant differences in TDFs between some temperature treatments within a diet quality treatment, these differences were no larger than approximately 0.2 ‰ for either carbon or nitrogen. Differences of less than 0.2 ‰ in TDFs across the range of tested temperatures is near the range of error in typical isotopic analyses and is generally not enough to make an ecologically meaningful difference in dietary modelling exercises [67].

Few studies have experimentally tested the effects of temperature on TDFs, and the results of those that have are somewhat equivocal. Barnes et al. [68] found that, in bass (*Dicentrarchus labrax)*, higher temperatures (18°C vs. 23°C) resulted in higher Δ^13^C values, but lower Δ^15^N values. Britton and Busst [14] found that both Δ^13^C and Δ^15^N values were slightly higher (~0.3–0.5 ‰) in some tissues among three fish species at a higher temperature. Bloomfield et al. [13], in contrast, found generally smaller TDFs at higher temperatures in black bream (*Acanthopagrus butcheri*). Each of these studies tested only two experimental levels of temperature, however, so the results of this study are likely a more sensitive estimation of temperature effects. Godiksen et al. [69] conducted a study of the effect of water temperature on TDFs in *Gadus morhua*. They tested four temperatures (4, 7, 10 and 14°C) and found no effect of temperature on Δ^13^C values in muscle and heart tissue, while Δ^15^N values decreased slightly (~0.6 ‰) with increasing temperature. Further studies isolating temperature as a variable in trophic discrimination and testing a wider range of temperatures across multiple species would help to disentangle the somewhat contradictory results seen thus far. Temperature effects on TDFs may ultimately be a taxa or species-specific parameter, with some species such as Pacific cod showing little TDF sensitivity to temperature and other species responding to a greater degree.

## Conclusions

The continued growth of studies employing stable isotope dietary mixing models reinforces the need for accurate, empirically derived data to be available for parameterizing these models [12]. The work contained herein advances the knowledge base available for dietary modelling in Pacific cod and in other related species and incorporates variability due to temperature and dietary quality. As global climate warming affects nearly every oceanic ecosystem in some fashion [70], climate-informed modelling parameters will be vital to predicting the effects of climate change on marine food webs, marine ecosystem structure and function, and the future health of global fisheries.

## Supporting information

S1 File Statistical models and results. Mixed-effects statistical model structure, model selection criteria, and post-hoc pairwise comparison results. Results are presented for each set of TDF data (nitrogen, carbon, and lipid-corrected carbon TDFs).
(XLSX)
